# Coinfection With SARS-CoV-2 and Dengue Virus: A Case Report Highlighting Diagnostic Challenges

**DOI:** 10.3389/fitd.2022.801276

**Published:** 2022-02-15

**Authors:** Prasetyo Hariadi, Dewi Lokida, Adhella Menur Naysilla, Nurhayati Lukman, Herman Kosasih, Yan Mardian, Gestana Andru, Inggar Pertiwi, Retna I. Sugiyono, Antonius A. Pradana, Gustiani Salim, Deni P. Butar-butar, Chuen-Yen Lau, Muhammad Karyana

**Affiliations:** 1 Tangerang District Hospital, Tangerang, Indonesia; 2 Indonesia Research Partnership on Infectious Disease (INA-RESPOND), Jakarta, Indonesia; 3 HIV Dynamics and Replication Program, Center for Cancer Research, National Cancer Institute, National Institutes of Health, Bethesda, MD, United States; 4 National Institute of Health Research and Development (NIHRD), Ministry of Health, Jakarta, Indonesia

**Keywords:** coinfection, dengue virus, SARS-CoV-2, serology, diagnostic challenge

## Abstract

**Background:**

Since its emergence in China, SARS-CoV-2 has infected more than 240 million people worldwide, including in regions where dengue virus (DENV) is hyperendemic such as Latin America and Southeast Asia, including Indonesia. Diagnosis of COVID-19 in dengue endemic regions as well as DENV and SARS-CoV-2 co-infection can be challenging.

**Case Presentation:**

We describe a 68-year-old woman with diabetes mellitus type II who was admitted to the Tangerang District Hospital on 14 April 2020. She lived in a neighborhood where a few people were contracting dengue fever. She presented with five days of fever, malaise, anorexia, nausea, myalgia, and arthralgia. Hematology revealed anemia, thrombocytopenia, normal leukocyte count, increased neutrophil proportion, and decreased lymphocyte proportion and absolute lymphocytes. Her chest X-ray showed right pericardial infiltrates. Although dengue was clinically suspected, she was also tested for SARS-CoV-2 infection as she met screening criteria. After being confirmed SARS-CoV-2 positive by RT-PCR, she was treated with ceftriaxone, paracetamol, azithromycin, oseltamivir, and chloroquine. She was clinically improved four days later and discharged from the hospital on 25 April 2020 after SARS-CoV-2 RT-PCR was negative on two consecutive samples. Dengue was diagnosed retrospectively based on sero-conversion of dengue IgM and a very high dengue IgG index (ELISA, Focus Diagnostics^®^, Cypress, CA, USA), and sero-conversion of dengue IgM and positive IgG (Rapid test, PanBio ^®^Dengue duo cassette, Inverness Medical Innovations, QLD, AU), which was equivalent to high Hemagglutination Inhibition (HI) antibody titer (≥1280) found in secondary dengue infection.

**Conclusion:**

The overlapping clinical presentations of COVID-19 and dengue; limited diagnostic capacity of laboratories in resource constrained settings; and complexities of interpreting results make identification of COVID-19 in the dengue endemic setting challenging. Clinicians in endemic areas must be aware of diagnostic challenges and maintain a high index of suspicion for COVID-19 coinfection with DENV and other tropical pathogens.

## BACKGROUND

Since its emergence at the end of December 2019 in Wuhan, China, SARS-CoV-2 has infected more than 240 million people worldwide, including in regions where dengue virus (DENV) is hyperendemic such as Southeast Asia and Latin America (1). Challenges associated with diagnosis of COVID-19 while DENV and other tropical infections are circulating and simultaneous infection by DENV and SARS-CoV-2 have been anticipated (2–7) as Indonesia is highly affected by both viruses. Since Indonesia’s first reported COVID-19 case in early March 2020, the number of confirmed cases has risen to >4.2 million with an estimated 3.4% mortality rate (8) Dengue, despite having a lower mortality rate (0.7%) than COVID-19, caused 108,000 cases in 2020 (9). We report the identification of DENV and SARS-CoV-2 coinfection in Indonesia to highlight the challenge of diagnosis when multiple tropical pathogens are circulating.

## CASE PRESENTATION

**FIGURE 1 |:**
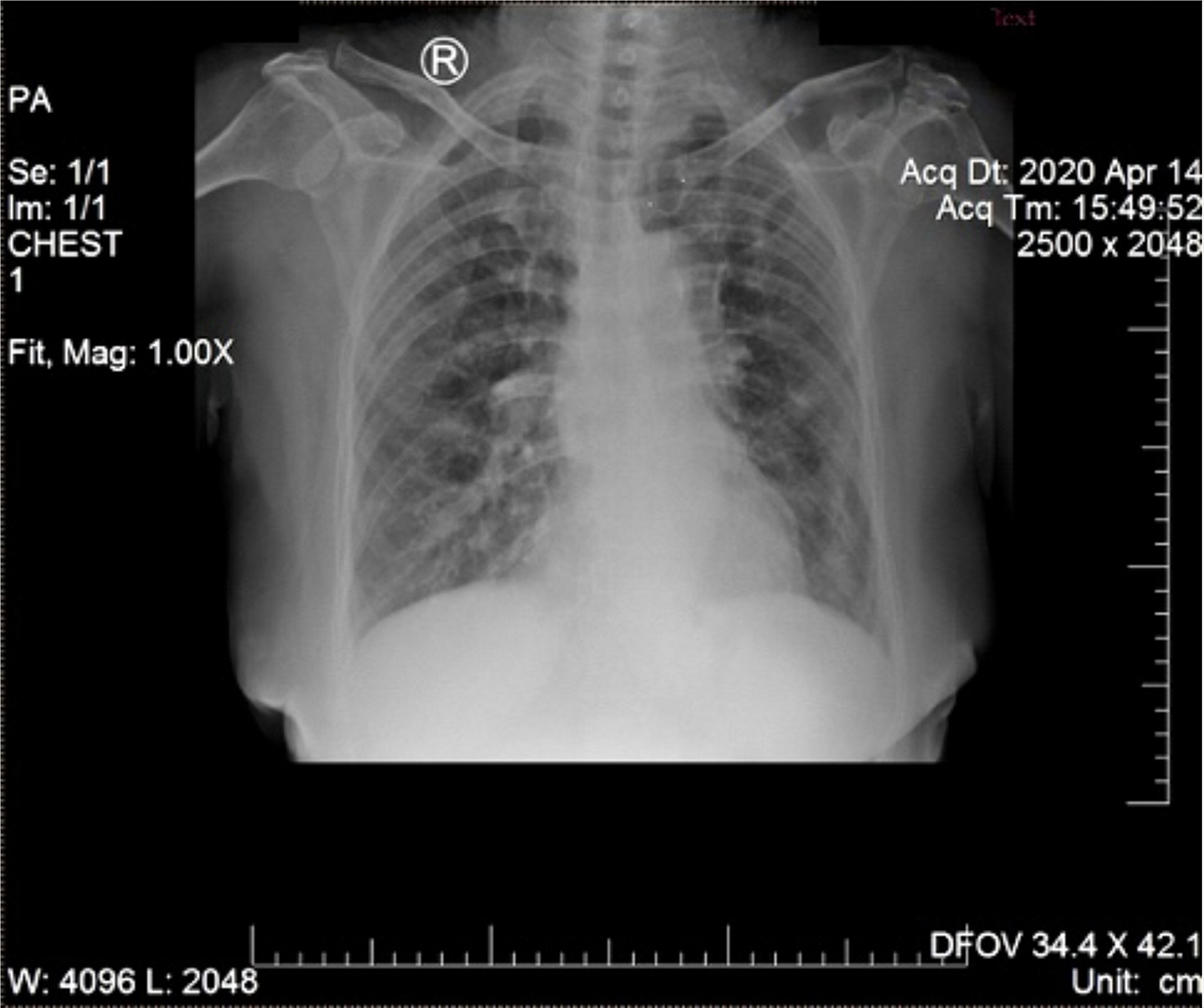
Chest X-ray on day of hospitalization. Pericardial infiltrates suggest bronchopneumonia.

A 68-year-old woman with chronic diabetes mellitus type II was referred to Tangerang District Hospital on 14 April 2020 by a general practitioner with a clinical diagnosis of dengue fever. She presented with five days of fever to 39°C managed with oral antipyretics, accompanied by malaise, anorexia, nausea, myalgia, and arthralgia. Cough and diarrhea had appeared one day later. She reported living in a neighborhood where a few people were contracting dengue fever and participating in a religious gathering four days prior to symptom onset. On exam, vital signs were within normal limits, except for mildly elevated blood pressure (150/90 mmHg). No rash or bleeding was observed. Chest X-ray (CXR) showed right pericardial infiltrates, suggesting bronchopneumonia (Figure 1). Hematology results revealed anemia (hemoglobin 10.9 mg/dL), thrombocytopenia (99,000/mm3), normal leukocyte count (8,000/mm3), increased neutrophil proportion (78%), decreased lymphocyte proportion (13%), and decreased absolute lymphocytes (1040/mm3). Her random blood sugar, ureum and creatinine levels were normal (110 mg/dL, 24 mg/dL, and 0.8 mg/dL, respectively). She was taking glibenclamide, and had no history of heart or kidney disease.

Despite agreement that the patient had dengue based on clinical presentation, she still underwent COVID-19 screening. Per national regulations, hospitals screen all patients for COVID-19 at admission and isolate them if the virus is suspected. Consultations with the internist and pulmonologist occurred because the patient met COVID-19 screening criteria (fever, pericardial infiltrates on CXR, and exposure history). A COVID-19 serologic rapid diagnostic test (RDT) was ordered and returned faintly positive for IgM and IgG antibody combined (Wondfo^®^, Wondfo Biotech, Guangzhou, China). The patient was then admitted to the isolation ward for supportive care.

Pending SARS-CoV-2 rRT-PCR results (10) from mixed nasopharyngeal and oropharyngeal specimens, the patient was empirically treated with ceftriaxone (1g IV q12hr for seven days) and paracetamol (500mg PO q8hr). SARS-CoV-2 infection was confirmed two days later based on the 15 April 2020 specimen, which showed Ct values of 29.9 and 31.1 for N1 and N2 genes, respectively. The pulmonologist treated her as moderate COVID-19 and administered azithromycin (500mg PO q24hr PO for three days), oseltamivir (75mg PO q12hr for five days), and chloroquine (250mg PO q12hr for five days) per the COVID-19 management guidelines from the Indonesian Society of Respirology and available evidence at the time (11, 12). As COVID-19 had been confirmed, laboratory work up for dengue (DENV IgM/IgG RDT) was not performed. The patient clinically improved (resolution of fever, improvement of constitutional symptoms and normal platelet count (189,000/mm^3^) by 18 April 2020. Follow-up SARS-CoV-2 PCR was negative on two consecutive tests 24 hours apart (21 and 22 April 2020). She was discharged from the hospital on 25 April 2020.

**FIGURE 2 |:**
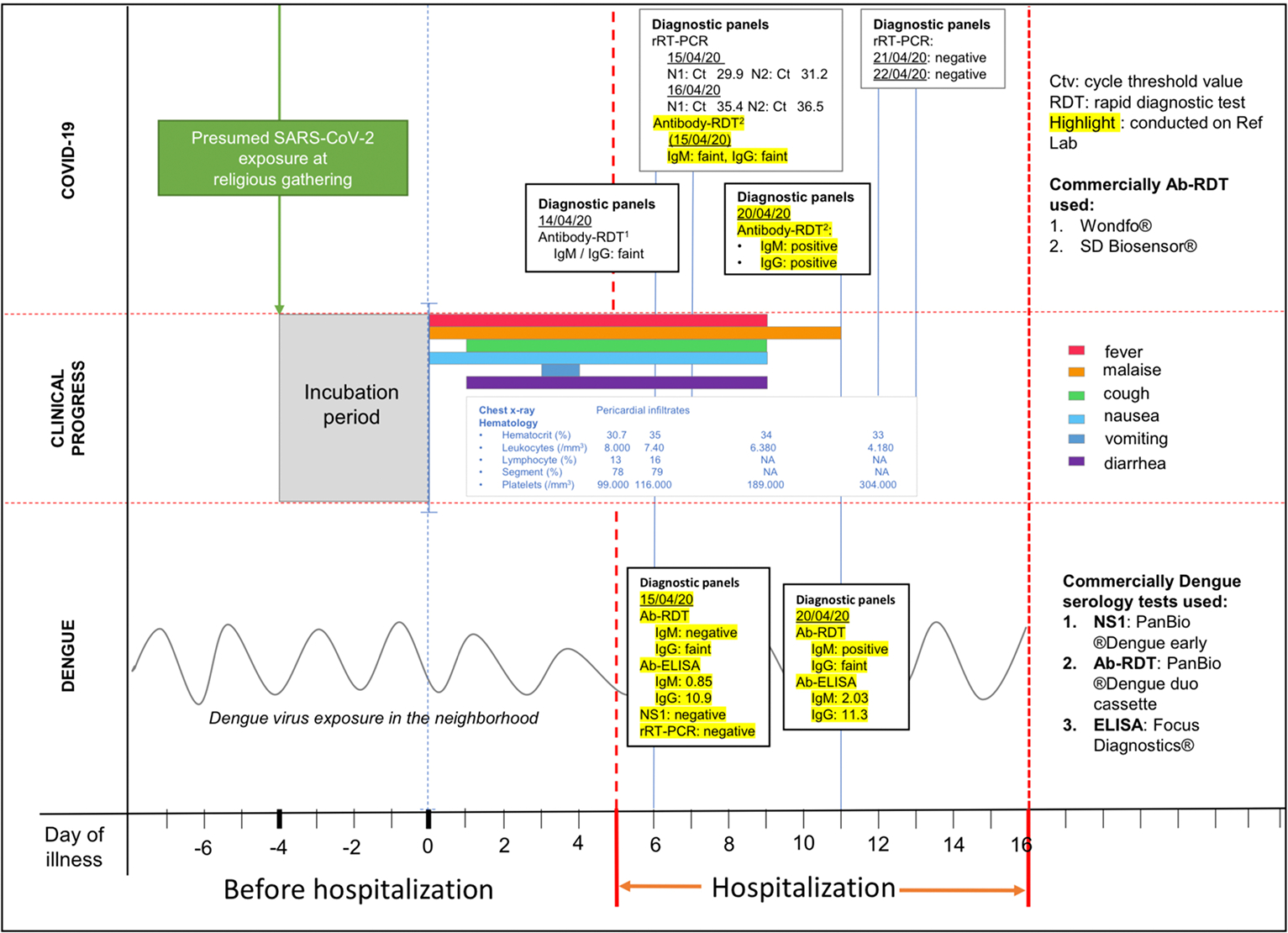
Time course of clinical and laboratory findings. COVID-19 data is shown in the top panel; clinical course is shown in the middle panel; dengue data is shown in the bottom panel. Procedures highlighted in yellow were performed retrospectively for research purposes.

Retrospective serology for SARS-CoV-2 IgM and IgG antibodies using RDT SD Biosensor, Korea) was performed in addition to PCR testing. The IgM and IgG bands appeared faint in acute serum (day 6 of illness) and were strongly positive in the convalescent serum (day 11). Dengue diagnostic tests were also performed retrospectively. The diagnosis of DENV infection was based on seroconversion of IgM (index values of 0.85 in acute to 2.03 in convalescent sera) by ELISA (Focus Diagnostics^®^, Cypress, CA, USA) and by rapid test (from negative to positive, PanBio ^®^ Dengue duo cassette, Inverness Medical Innovations, QLD, AU) and the positive IgG in acute and convalescent sera by rapid test (PanBio ^®^Dengue duo cassette, Inverness Medical Innovations, QLD, AU). This was confirmed by high index (11.3) values of IgG ELISA (Focus Diagnostics^®^, Cypress, CA, USA). The IgG detection threshold for this rapid immunochromatography test is set at a high IgG titer, equivalent to HI titer of ≥1280. Thus, positive rapid IgG test in both acute and convalescent sera was considered indicative of acute secondary dengue infection. DENV NS1 (PanBio ^®^Dengue early, Inverness Medical Innovations, QLD, AU) and DENV rRT-PCR from acute serum were negative. All serologic assays were performed in the Reference Laboratory of INA-RESPOND. Clinical course and laboratory results are shown in Figure 2.

## DISCUSSION AND CONCLUSION

We report a SARS-CoV-2 and DENV co-infection that occurred in April 2020, at the beginning of the pandemic in Indonesia. Since then, several additional co-infections have been recognized (13, 14). Our patient’s clinical manifestations were moderate despite having dual DENV and SARS-CoV-2 infections on the backdrop of DM type II. This may be attributable to well-controlled blood sugar, the absence of DM related complications such as heart or kidney diseases (15), and possibly the rapid clearance of DENV in blood during recurrent infection. In recurrent DENV infection, IgG antibodies rise quickly during acute illness, often in the absence or with low titers of IgM antibodies (16). This may explain the negative DENV RT-PCR and NS1. It has been reported that DENV in secondary infection peaks on day 2 of illness and then decreases rapidly to undetectable on day 5 (17). Similarly, NS1 in secondary infection disappears earlier than in primary infection and is often undetected (17–19). Although potential cross-reactivity between SARS-CoV-2 and DENV antibodies in rapid serological tests has been suggested (13, 20–23), recent dengue virus infection in our case was confirmed by seroconversion in paired acute-convalescent sera using both RDT and ELISA to exclude a false-positive dengue serology. Our patient’s conversion to SARS-CoV-2 PCR negativity by day 12 of illness is within the range expected (24, 25). However, we do not know precisely when SARS-CoV-2 cleared as we did not have swabs between day seven (positive) and day 12 (negative).

The overlapping clinical presentations of COVID-19, dengue and typhoid fever; limited diagnostic capacity of laboratories in resource constrained settings; and complexities of interpreting results make identification of COVID-19 in the dengue endemic setting challenging. Constitutional symptoms, such as fever, headache, and myalgia are often reported in both symptomatic dengue infection and during COVID-19 (26, 27). Both diseases can present with thrombocytopenia associated with depressed platelet synthesis due to virus-induced bone marrow suppression and immune-mediated clearance of platelets (26). However, clinical manifestations diverge as the COVID and dengue progress. The hallmark of dengue infection is progression to plasma leakage, contracted intravascular volume, and hypovolemic shock in the critical phase (28). COVID-19 can progress to severe pneumonia with acute respiratory distress syndrome, which can lead to respiratory failure, septic shock, and/or multiple organ dysfunction (29).

Dengue can be clinically diagnosed and confirmed by assays including anti-DENV antibodies, non-structural protein 1 (NS1) antigen, or DENV-specific nucleic acid detection (30). Dengue NS1 and IgM/IgG RDTs are useful in the emergency room to guide clinicians. However, several studies in Indonesia have reported detection of dengue antibodies in confirmed COVID-19 patients (13, 21, 23, 31), and cross-reactivity between DENV and COVID-19 antibody RDTs has been suggested based on similarities between epitopes in the HR2 domain of the SARS-CoV-2 spike protein and the dengue envelope protein from in-silico analysis, though false positives and alternate antigen cross-reactivity cannot be ruled out (32). Cross-reactivity between Covid-19 antibodies and other co-circulating infections is also possible (33). Continued development of diagnostic tests, such as reverse transcriptase loop-mediated isothermal amplification (RT-LAMP), CRISPR, and antigen-based assay RDTs, is essential for identification of COVID-19 cases (34, 35).

When our case occurred, RDTs for the SARS-CoV-2 antigen were not available, as reflected by the assessments of the general practitioner and emergency room physician, both of whom suspected dengue based on clinical presentation and ongoing local transmission of DENV. Along with a high index of suspicion, epidemiology, clinical symptoms, laboratory testing, and imaging enable diagnosis of COVID-19 (27). Radiography, including CXR, is performed to screen suspected cases according to the COVID-19 diagnosis and management guidelines from Indonesian Society of Respirology. CXR findings of multiple bilateral ground-glass opacities in the peripheral lower lung zone and pericardial infiltrate may help distinguish COVID-19 from dengue, the latter of which may present with pleural effusions (34, 36). Subsequently, the pulmonologist suspected COVID-19 despite the existing DENV clinical diagnosis in light of the consistent respiratory, laboratory, and imaging findings. It was then discovered that the patient had contact with a confirmed COVID-19 case 4 days prior to emergence of symptoms.

Despite reports of low sensitivity of the COVID-19 antibody RDT during acute illness (37), use in this patient was vital as it facilitated her management as a COVID-19 patient. Difficulty in distinguishing COVID-19 and dengue, particularly when diagnostics perform suboptimally, has been reported in other countries including Singapore (20) and Thailand (38). Findings from a tertiary hospital in Singapore indicate that routine screening of patients with viral prodromes during a dual outbreak of COVID-19 and dengue can enable containment of COVID-19 cases masquerading as dengue (39). Nowadays, presence of SARS-CoV-2 antigen based RDTs with excellent performance within the first week of illness has largely replaced rapid antibody tests for early detection of COVID-19, especially in resource-limited settings (34). Furthermore, positive dengue infection should not prevent evaluation for COVID-19, particularly when patients have a history of contact with suspected or confirmed COVID-19 cases.

At the time of this co-infection, the standard of care for both dengue and COVID-19 in Indonesia was supportive care. No effective specific antivirals for Dengue or COVID were yet available. Thus, clinical management of the patient would not have been altered by knowledge of the diagnosis. However, unrecognized COVID-19 presents epidemiological risks, particularly the possibility of transmission to hospital staff and other patients. This patient’s household contacts were tested for SARS-CoV-2 IgM and IgG using RDT and found to be negative. Ideally molecular diagnosis and appropriate isolation precautions would be employed at presentation for patients at risk for COVID-19. With improving COVID-19 treatment options ranging from antivirals, passive immunotherapy, anti-inflammatory drugs, and cell-based therapy (40), early diagnosis can facilitate better outcomes. Furthermore, clinicians must be cognizant of the possibility of SARS-CoV-2 infections amongst those who are vaccinated or have been previously infected.

Diagnostic strategies specifically for resource limited settings in which health care systems are already overburdened are needed. Point-of-care RDT antigen tests will expedite triage of patients due to sensitivity in detecting high pathogen load associated with the early phase of infection (41). Combination RDT antigen and RDT antibody tests could improve COVID-19 detection in patients presenting to the emergency department (42). Continued development of preventive and therapeutic approaches for both DENV and COVID-19 will also be helpful for alleviating risks to patients as well as burden on the healthcare system. In the meantime, clinicians in endemic areas must maintain a high index of suspicion for the concurrent infection with COVID-19, DENV, and other tropical pathogens.

## Data Availability

The original contributions presented in the study are included in the article/supplementary material. Further inquiries can be directed to the corresponding author.
